# IGCNSDA: unraveling disease-associated snoRNAs with an interpretable graph convolutional network

**DOI:** 10.1093/bib/bbae179

**Published:** 2024-04-21

**Authors:** Xiaowen Hu, Pan Zhang, Dayun Liu, Jiaxuan Zhang, Yuanpeng Zhang, Yihan Dong, Yanhao Fan, Lei Deng

**Affiliations:** School of Computer Science and Engineering, Central South University, 410075, Changsha, China; Hunan Provincial Key Laboratory of Clinical Epidemiology, Xiangya School of Public Health, Central South University, 410078, ChangshaChina; School of Computer Science and Engineering, Central South University, 410075, Changsha, China; Department of Electrical and Computer Engineering, University of California, San Diego, 92093, CA, United States; School of Software, Xinjiang University, 830046, Urumqi, China; School of Computer Science and Engineering, Central South University, 410075, Changsha, China; School of Computer Science and Engineering, Central South University, 410075, Changsha, China; School of Computer Science and Engineering, Central South University, 410075, Changsha, China

**Keywords:** snoRNA–disease association, graph convolutional network, interpretability analysis, subgraph

## Abstract

Accurately delineating the connection between short nucleolar RNA (snoRNA) and disease is crucial for advancing disease detection and treatment. While traditional biological experimental methods are effective, they are labor-intensive, costly and lack scalability. With the ongoing progress in computer technology, an increasing number of deep learning techniques are being employed to predict snoRNA–disease associations. Nevertheless, the majority of these methods are black-box models, lacking interpretability and the capability to elucidate the snoRNA–disease association mechanism. In this study, we introduce IGCNSDA, an innovative and interpretable graph convolutional network (GCN) approach tailored for the efficient inference of snoRNA–disease associations. IGCNSDA leverages the GCN framework to extract node feature representations of snoRNAs and diseases from the bipartite snoRNA-disease graph. SnoRNAs with high similarity are more likely to be linked to analogous diseases, and vice versa. To facilitate this process, we introduce a subgraph generation algorithm that effectively groups similar snoRNAs and their associated diseases into cohesive subgraphs. Subsequently, we aggregate information from neighboring nodes within these subgraphs, iteratively updating the embeddings of snoRNAs and diseases. The experimental results demonstrate that IGCNSDA outperforms the most recent, highly relevant methods. Additionally, our interpretability analysis provides compelling evidence that IGCNSDA adeptly captures the underlying similarity between snoRNAs and diseases, thus affording researchers enhanced insights into the snoRNA–disease association mechanism. Furthermore, we present illustrative case studies that demonstrate the utility of IGCNSDA as a valuable tool for efficiently predicting potential snoRNA–disease associations. The dataset and source code for IGCNSDA are openly accessible at: https://github.com/altriavin/IGCNSDA.

Small nucleolar RNAs (snoRNAs) represent a pivotal group of non-coding RNAs found in eukaryotic cells, generally spanning from 60 to 300 nucleotides in length. Their primary residence is within the nucleolus. In terms of their functional and structural classification, snoRNAs can be primarily grouped into two categories: box C/D snoRNAs and box H/ACA snoRNAs. These categories are responsible for orchestrating the site-specific 2’-O-ribose methylation of rRNA [1] and the pseudouracil modification of rRNA [2]. Beyond rRNA, snoRNAs influence tRNA methylation [3, 4] and even guide mRNA alternative splicing [5]. Many snoRNAs are chiefly located within intronic regions of genes transcribed by RNA polymerase II. However, snoRNAs can also originate from intronic regions of long noncoding RNAs (lncRNAs). Guided by internal signal sequences, snoRNAs associate with specific proteins to form snoRNP complexes [6]. This association safeguards against enzymatic cleavage and facilitates involvement in RNA biosynthesis, transport and function. Over the course of history, snoRNAs were initially regarded as mere transcriptional byproducts; however, the application of advanced sequencing techniques has unveiled their multifaceted functionalities. snoRNAs are now recognized to play pivotal roles in disease-related biological processes. For instance, snoRNA HBII-52 participates in serotonin receptor 2C alternative splicing, influencing Prader–Willi syndrome onset [7]. SNORD78 correlates with poor prognosis in Non-small-cell lung cancer; elevated SNORD78 expression is linked to worse outcomes compared with low-expression patients [8]. Breast cancer tumors show notable down-regulation of SNORD46 and SNORD89 relative to normal tissue samples [9]. SNORA80E is notably overexpressed in distinct colorectal and lung cancer signatures [10, 11]. While considerable research underscores the strong link between snoRNAs and disease, a comprehensive understanding of their impact on human health remains elusive. On one front, identifying potential snoRNA–disease associations could yield diagnostic markers; on another, it could enhance comprehension of intricate pathogenic mechanisms. The tally of snoRNA–disease associations gleaned from biological experiments is consistently growing. Public-access databases such as RNADisease v4.0 [12] and ncRPheno [13] have surfaced. Given the substantial temporal, labor and financial investments in biological experiments, the use of computational methods to predict new associations between snoRNAs and diseases from these databases has become a thriving research field. Computational approaches, compared with conventional biological experimentation, offer swifter, more efficient and more scalable means.

In the contemporary landscape, there exists a rapid advancement in deep learning technology. Concurrently, an increasing number of researchers are endeavoring to employ computational methodologies for addressing forefront challenges [14], with a particular emphasis on the prediction of associations between RNA and diseases, such as lncRNA-disease [15–19], miRNA-disease [20, 21], circRNA-disease [22–24], piRNA-disease [25, 26] and snoRNA-disease [27]. A method called VGAELDA [15] integrates variational graph autoencoders and graph autoencoders and applies a variational expectation-maximization algorithm for training to predict lncRNA–disease associations. IPCARF [16] employs a fusion of various similarity metrics and melds incremental principal component analysis with random forest techniques to forecast previously uncharted lncRNA–disease connections. LDGRNMF [17] initially leverages a gaussian interaction profile kernel along with disease semantic data for estimating disease similarity. Subsequently, it employs weighted k-nearest neighbor interaction profiles to predict potential associations between lncRNAs and diseases. A novel approach, GCRFLDA [18], leverages a combination of graph convolution matrices, attention mechanisms and conditional random fields to unveil previously undiscovered connections between lncRNAs and diseases. Chen *et al.* [20] introduced the DBNMDA model, a deep belief network-based framework designed for predicting potential associations between miRNAs and diseases. This innovative approach leverages the entirety of available miRNA–disease pairs information, effectively addressing the limitations of scarce known associations. Zhang *et al.* [21] proposed NIMGSA, a computational approach that integrates graph autoencoders and the self-attention mechanism. NIMGSA strategically combines inductive matrix completion with label propagation, enhancing the predictive performance of association predictions. Lei *et al.* [22] conducted an extensive investigation into the associations between circRNA–miRNA and miRNA–disease interactions, culminating in the construction of a comprehensive circRNA–disease pairing network. Subsequently, they employed the LeaderRank algorithm to effectively address the issue of inadequate negative data, thereby enhancing the quality of their analysis. The CDWBMS method, as introduced by Lei *et al.* [23], employs weighted meta-paths within a heterogeneous network for the purpose of predicting potential circRNA–disease associations. A computational method named GCNCDA [24] applies FastGCN to capture latent high-level features through the amalgamation of circRNA, diseases and their established associations. The Forest PA is then used to predict new circRNA–disease associations. A model called DFLPiDA [25] considers the features of piRNA and disease as images. Accordingly, it utilizes the convolutional denoising autoencoder to learn high-level features of piRNA–disease similarities for piRNA–disease association prediction. A model named SPRDA [26] uses the structural perturbation method to discover potential piRNA–disease associations. While the aforementioned methods have yielded commendable prediction outcomes, the association prediction models employed are essentially black box models, devoid of interpretability. Consequently, they fail to offer a rational explanation for prediction results from a biochemical standpoint.

To address the above issues, we introduce an interpretable method, IGCNSDA, which is based on GCN, to predict potential associations between snoRNAs and diseases. The key concept behind IGCNSDA is as follows. In our study, we commence by employing a GCN methodology to delineate the underlying relationships between snoRNAs and diseases in the bipartite snoRNA–disease graph. Subsequently, we implement a subgraph generation algorithm to partition similar snoRNAs and their associated diseases into distinct subgraphs. Within these subgraphs, we collate information from adjacent nodes and iteratively refine the representations of snoRNAs and diseases. Subsequently, we apply a layer aggregation technique to derive the ultimate embeddings for snoRNAs and diseases. Lastly, we leverage the inner product to predict association scores between snoRNAs and diseases. Our extensive experimentation and independent validation substantiate the superior performance of IGCNSDA over current state-of-the-art methodologies. Furthermore, interpretability analysis and case studies underscore IGCNSDA as an invaluable tool for predicting potential snoRNA–disease associations, which can guide biological experiments effectively.

## 1 MATERIALS AND METHODS

### 1.1 Dataset

In our research, we sourced our benchmark dataset from RNADisease v4.0 [12], a comprehensive repository that consolidates both experimentally validated and predicted associations between non-coding RNAs (ncRNAs) and diseases, derived from various authoritative sources in the literature. Specifically, we focused on snoRNA–disease associations by querying the RNADisease dataset. Careful curation efforts were then undertaken to remove redundant and incomplete entries, resulting in a refined benchmark dataset comprising 471 snoRNAs, 84 diseases and a total of 1095 well-documented associations among these entities. After that, we randomly divided the associated pairs into a training set and a test set at a ratio of 8:2. Finally, the training set contained 876 associated pairs and the test set contained 219 associated pairs. In addition, in order to comprehensively evaluate the performance of IGCNSDA, we selected the ncRPheo dataset [13] for independent testing. We obtain the data from the ncRPheo database and remove the data that already exists in RNADisease. Finally, we obtained 439 associations between 82 diseases and 13 snoRNAs for testing and used all RNADisease data for training. Finally, the data related to RNADisease and independent testing are shown in Table 1.

**Table 1:** Data distribution for the RNADisease dataset and independent tests

	RNADisease	ncRPheo
No. of snoRNA	471	13
No. of disease	84	82
No. of association	1095	439

To enhance accessibility, we express the associations between snoRNAs and diseases as a binary matrix, labeled as $SD$. A value of 1 is assigned to the corresponding element in the $SD$ matrix if a given snoRNA is linked to a specific disease; otherwise, it is set to 0.

### 1.2 The IGCNSDA framework

**Figure 1:**
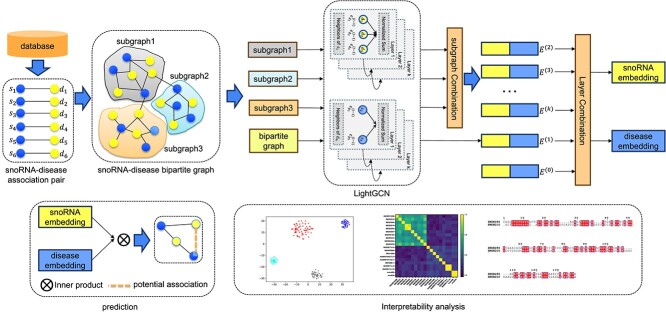
The overview of IGCNSDA. IGCNSDA takes snoRNA–disease association pairs as input and produces specific snoRNA–disease association scores. Initially, IGCNSDA constructs a bipartite graph representing snoRNA–disease associations. In this bipartite graph, LightGCN is employed to capture first-order associations. Subsequently, a subgraph generation algorithm is applied to create various subgraphs within the snoRNA–disease bipartite graph. Specifically, we frame subgraph generation as a node multi-classification problem. For each snoRNA, a two-layer MLP is utilized to categorize the snoRNA and all associated diseases into a subgraph. Within each subgraph, LightGCN is employed to capture high-order associations. Ultimately, the final embeddings of snoRNA and disease are computed by averaging the embeddings from each layer. The association score for a specific snoRNA–disease pair is determined using the inner product.

Figure 1 illustrates the core components of IGCNSDA, which comprises three main stages. Firstly, IGCNSDA employs a GCN to initially investigate first-order connections between snoRNA and disease nodes within the bipartite snoRNA–disease graph. Subsequently, IGCNSDA employs a subgraph generation algorithm to partition snoRNA nodes into distinct subgraphs. Within these isolated subgraphs, it aggregates information from neighboring nodes to reveal higher order relationships between snoRNA and disease. Following this, by aggregating the results from each GCN layer, it obtains the final embedding representation for snoRNA–disease associations. Lastly, an inner product is applied to compute association scores between snoRNA and disease, aiding in the prediction of potential associations between them.

#### 1.2.1 SnoRNA–disease bipartite graph

Based on our previously constructed association matrix $SD$, We can explicitly build the known snoRNA–disease associations as a snoRNA–disease bipartite graph $G=\left (V_{r}, V_{d}, E\right )$: we treat each snoRNA and disease as a node in the bipartite graph, where $V_{r}$ and $V_{d}$ represent the sets of snoRNAs and diseases, respectively. For each element that values 1 in matrix $SD$, we construct an edge $e$ between the corresponding snoRNA node and disease node, which represents that there is a valid interaction between these two different kinds of nodes. We employ the bipartite graph $G$ to serve as the input for IGCNSDA, facilitating the embedding enhancement for snoRNA and disease.

#### 1.2.2 Initialize embedding

In this section, we initialize an embedding lookup table for snoRNA and disease, respectively. With these tables, we can map each ID one-hot vector of snoRNA or disease to a relatively dense embedding representation through embedding lookup. Specifically, for $N$ one-hot snoRNA vectors and $M$ one-hot disease vectors, the embedding lookup tables can be described as 


(1) \begin{align*}& \left\{\begin{aligned} W_{r}=[e_{r1}^{0}, e_{r2}^{0}, \ldots, e_{rN}^{0}] \\ W_{d}=[e_{d1}^{0}, e_{d2}^{0}, \ldots, e_{dM}^{0}] \\ \end{aligned}\right.,\end{align*}

where $W_{r}$ and $W_{d}$ denote the embedding lookup table for snoRNAs and diseases, respectively; $e_{ri}^{0}\in R^{L}$ denotes the ID embedding of $i$th snoRNA and $e_{dj}^{0}\in R^{L}$ denotes the ID embedding of $j$th disease, where $L$ is the size of each ID embedding. In particular, for snoRNA $r$ and disease $d$, we specify their ID embeddings as $e_{r}^{0}$ and $e_{d}^{0}$, respectively.

#### 1.2.3 First-order embedding propagation

Similar to some commonly used GCN-based models [28, 29], IGCNSDA uses graph convolution to extract historical association information between snoRNA and disease to improve the embedding learning of each node. Since the direct associations between snoRNAs and diseases provide the most valid interactive information, which matters the most to the refinement of embedding representations, all the direct neighbors are put to participate in the first-order neighborhood aggregation. Notice that $e_{r}^{0}$ and $e_{d}^{0}$ are simply derived from their ID features, the embedding itself does not possess rich semantic attributes. Inspired by LightGCN [29], we exclusively retain the neighbor node aggregation operation within the graph convolutional network (GCN). The first-order neighborhood aggregation is shown as below 


(2) \begin{align*}& \left\{\begin{aligned} e_{r}^{1}=\sum_{d \in N_{r}} \frac{1}{\sqrt{\left|N_{r}\right|} \sqrt{\left|N_{d}\right|}} e_{r}^{0} \\ e_{d}^{1}=\sum_{r \in N_{d}} \frac{1}{\sqrt{\left|N_{r}\right|} \sqrt{\left|N_{d}\right|}} e_{d}^{0} \\ \end{aligned}\right.,\end{align*}

where $e_{r}^{1}$ and $e_{d}^{1}$ denote the first-order embeddings of snoRNA $r$ and disease $d$, respectively; $N_{r}$ and $N_{d}$ denote the sets of diseases interacting with snoRNA $r$ and snoRNAs interacting with disease $d$, respectively. The term $\frac{1}{\sqrt{|N_{r}|}\sqrt{|N_{d}|}}$ corresponds to Laplace normalization.

### 1.3 Subgraph generation algorithm

Based on the similarity of snoRNAs, it is more likely that they are associated with similar diseases, and vice versa. In this regard, we propose a subgraph generation algorithm that categorizes similar snoRNAs and their associated diseases into a subgraph. In terms of the results, each subgraph contains similar snoRNAs and their associated diseases. Therefore, we formalize this as a multi-classification task [30], assigning each snoRNA to different subgraphs. Let $G_{s}$ represent a subgraph, where $s \in \{1, 2, \cdots , N_{s}\}$ and $N_{s}$ is the total number of subgraphs.

After the first-order neighbor aggregation operation, we can obtain a first-order feature vector for each snoRNA. Subsequently, we perform feature fusion by combining the first-order feature vector with the initial feature vector of each snoRNA, resulting in the fused vector for each snoRNA as follows: 


(3) \begin{align*} F_{r} = LeakyReLU(W_{1}(e_{r}^{(0)} + e_{r}^{(1)}) + b_{1}),\end{align*}

where $F_{r}$ denotes the feature vector of snoRNA $r$ after the feature fusion operation. $W_{1}\in R^{d\times d}$ is the trainable weight matrix of the fusion operation and $b_{1}\in R^{1\times d}$ is the corresponding bias vector. As for the activation function of our model, we utilized LeakyReLU. To find out which subgraph snoRNA $r$ belongs to, we take $F_{r}$ as the input and use a 2-layer MLP to generate its prediction vector 


(4) \begin{align*}& \left\{\begin{aligned} H=\text{LeakyReLU}(W_{2} F_{r} + b_{2}) \\ O=W_{3} H+b_{3}, \\ \end{aligned}\right.\end{align*}

where $O$ denotes the prediction vector. We take the index where the maximum value in $O$ is located as the number of the subgraph to which the snoRNA $r$ belongs. Notice that the dimension of the prediction vector needs to be exactly the same as the number of subgraphs, which we set as a hyperparameter.

### 1.4 High-order embedding propagation

Within each subgraph, we aggregate information from neighboring nodes to enhance the embeddings learned by similar snoRNAs, thereby increasing their similarity. After introducing the subgraph generator module, we notice that each snoRNA node only exists in one subgraph, and all disease nodes that interact with it also exist in this subgraph. Therefore, it remains unchanged that all snoRNA nodes can still aggregate all their original neighbor nodes to update their embedding representations. In comparison, a disease node can exist in multiple subgraphs since its first-order snoRNA neighbors may be scattered in more than one subgraph. Accordingly, for each subgraph containing disease $d$, we generate an embedding for $d$ separately. We represent the embedding of disease $d$ after $k$ layers of GCN within subgraph $r$ as $e_{ds}^{k}$. The calculation formulas of high-order neighborhood aggregation in IGCNSDA are defined as 


(5) \begin{align*}& \left\{\begin{aligned} e_{r}^{k+1} &=\sum_{d s \in N_{r}} \frac{1}{\sqrt{\left|N_{r}\right|} \sqrt{\left|N_{ds}\right|}} e_{d s}^{k} \\ e_{d s}^{k+1} &=\sum_{r \in N_{ds}} \frac{1}{\sqrt{\left|N_{ds}\right|} \sqrt{\left|N_{r}\right|}} e_{r}^{k} \\ \end{aligned} \right.,\end{align*}

where $N_{ds}$ represents the set of snoRNAs which interact with disease $d$ within subgraph $s$. These formulas ensure that every node in a subgraph only propagates information within this specific subgraph. Since each subgraph consists of snoRNAs with similar interaction features and their directly associated disease items, this propagation way cuts off the introduction of latent noise from irrelevant high-order information, thus reducing unwanted interference to the learning of node embeddings. Consequently, this decelerates the convergence of node embeddings to an exceptionally indistinguishable state, ultimately enhancing the predictive capabilities of our model. This feature distinguishes IGCNSDA from previous GCN-based prediction models. In the context of layer $k$, we consolidate the embeddings of disease $d$ from all the subgraphs in which it is present, resulting in the ultimate representation of disease $d$


(6) \begin{align*}& e_{d}^{k}=\sum_{s \in S_{d}} e_{d s}^{k}.\end{align*}

Here, $S_{d}$ represents the collection of subgraphs containing disease $d$.

### 1.5 Layer combination

After aggregating information from a total of $K$ layers in the neighborhood, we generate the ultimate representations for snoRNA $r$ and disease $d$ by integrating their embeddings from each layer individually. This is expressed as follows: 


(7) \begin{align*}& \left\{\begin{aligned} e_{r}=\frac{1}{K + 1}\sum_{k=0}^{K} e_{r}^{k} \\ e_{d}=\frac{1}{K + 1}\sum_{k=0}^{K} e_{d}^{k} \\ \end{aligned}\right.,\end{align*}

Here, $k$ represents the current layer number.

At last, to determine the association between a given snoRNA $r$ and disease $d$, we calculate their similarity by computing the dot product of the final embeddings of snoRNA $r$ and disease $d$


(8) \begin{align*}& \widehat{y}_{r d}=e_{r}^{T} e_{d}.\end{align*}

### 1.6 Optimization

IGCNSDA treats each snoRNA’s final association prediction task as a top-n ranking recommendation for the most likely associated diseases. We employed the Bayesian Personalized Ranking Loss (BPR) [31, 32] as the loss function in our model training process. BPR is a pairwise loss function that encourages increasing the similarity between the embeddings of snoRNA and their actual interaction target disease embeddings. Our loss function formula is as follows: 


(9) \begin{align*}& L=\sum_{\left(r, d^{+}, d^{-}\right) \in T}-\ln \sigma\left(\widehat{y}_{r d^{+}}-\widehat{y}_{r d^{-}}\right)+\lambda\|\Theta\|_{2}^{2},\end{align*}

where $T$ represents the training set. Triplet $\{ r,d^{+},d^{-} \}$ is an instance of $T$, while snoRNA–disease pair $(r,d^{+})$ denotes an experimentally verified association and $(r,d^{-})$ denotes an unverified one. Our purpose is to make $\widehat{y}_{r d^{+}}$ scores higher than $\widehat{y}_{r d^{-}}$. $\Theta $ denotes parameters of our model, while $\lambda $ controls the strength of $L_{2}$ regularization.

Lastly, we outline the algorithm flowchart process for IGCNSDA in Algorithm 1. 


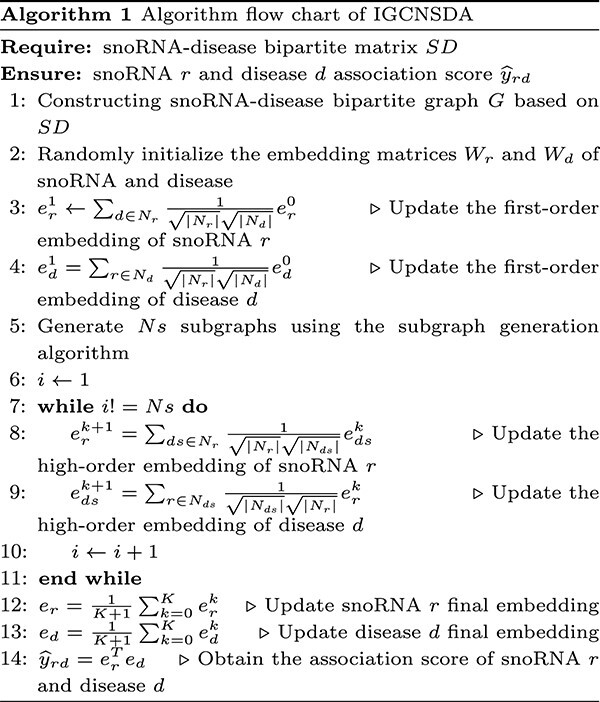


## 2 RESULTS

### 2.1 Experiment setup

To assess the robustness of the IGCNSDA, we conducted model performance evaluations through a 5-fold cross-validation approach. The dataset, which encompasses associations between snoRNAs and diseases, was randomly partitioned into five subsets. One of these subsets was designated as the test set, while the remaining four subsets were systematically employed for training in a rotating manner until each subset had served as the test set. The ultimate prediction results were derived by averaging the outcomes obtained from these five iterations. To assess the effectiveness of IGCNSDA, we employed two well-established metrics: AUC, AUPR and accaracy [33, 34]. AUC measures the classifier’s ability to distinguish between positive and negative samples, with higher values indicating superior performance. Conversely, AUPR quantifies the balance between precision and recall, providing a comprehensive evaluation of IGCNSDA’s predictive capabilities. Accuracy denotes the ratio of correctly classified samples to the total number of samples.

### 2.2 Comparative experiment

#### 2.2.1 Comparison with highly related methods

To evaluate the model’s performance, we conducted a comparative analysis against five closely related association prediction methods, as outlined below:

- **NIMCGCN** [35] employs GCN to acquire latent feature representations for miRNA and diseases based on similarity networks. Following this, the acquired characteristics are input into an innovative Neural Inductive Matrix Completion model, resulting in the generation of a finalized association matrix.
- **AMHMDA** [36] represents an innovative approach, harnessing attention-aware multi-view similarity networks in conjunction with hypergraph learning techniques. It leverages GCN to construct multiple similarity networks, introduces hypernodes and incorporates the attention mechanism to predict potential miRNA–disease associations.
- **NSAMDA** [37] involves the integration of miRNA sequence similarity and integrated similarity data to enhance miRNA features, followed by the creation of a heterogeneous miRNA–disease graph using these enriched features. Subsequently, a graph attention network-based neighbor selection method identifies the most significant neighbors for aggregation, and an inner product decoder is used to score miRNA–disease pairs.
- **iPiDA_GCN** [38] serves as a computational technique in this study, aimed at discerning piRNA–disease associations by harnessing the capabilities of GCN. It effectively extracts unique features from both piRNAs and diseases while leveraging association patterns within various networks. Through the utilization of fully connected networks and inner product calculations, iPiDA_GCN proficiently predicts scores for piRNA–disease associations.
- **VGAMF** [39] integrates various types of information about miRNAs and diseases into comprehensive similarity networks, derives nonlinear representations using variational graph auto-encoders, conducts non-negative matrix factorization on the miRNA–disease association matrix for linear representations, and employs a fully connected neural network to generate final predicted association scores for all miRNA–disease pairs.

**Figure 2:**
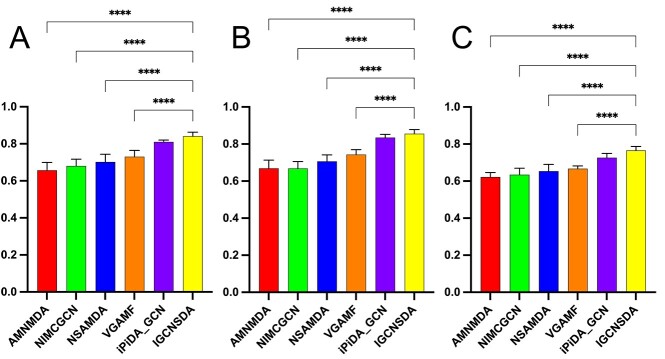
The results of comparative experiment on the RNADisease dataset. For (**A**), (**B**) and (**C**), ****$P$ <0.0001.

Table 2 showcases the outcomes of our IGCNSDA model, alongside five established methodologies. Each approach employs a 5-fold cross-validation technique [40] to derive the mean as the ultimate predictive outcome. Significantly, our proposed IGCNSDA model outperforms all others, with an average AUC of 0.8438, an average AUPR of 0.8744 and an average accuracy 0.7831. These figures reflect a substantial enhancement of 3.37%, 5.71% and 7.77%, respectively, compared with the suboptimal approach. We constructed histograms (Figure 2) for the respective datasets and applied one-way ANOVA to evaluate statistical differences in AUC, AUPR and accuracy during the 5-fold cross-validation of IGCNSDA and the remaining five methods. Subsequently, a Dunnett’s multiple comparisons posttest was conducted. Notably, in comparison with IGCNSDA, four of the methods exhibited statistically significant differences, with the exception of iPiDA_GCN. This more robustly substantiates the superiority of the IGCNSDA method in predicting the association between snoRNA and diseases. These empirical results robustly validate the effectiveness of IGCNSDA in predicting novel snoRNA–disease associations. The outstanding performance of IGCNSDA can be ascribed to its utilization of GCN in acquiring representations for snoRNAs and diseases, leveraging the rich neighborhood information embedded in the bipartite graph connecting snoRNAs and diseases. Additionally, IGCNSDA’s inventive subgraph generation algorithm streamlines the effective clustering of akin snoRNAs, empowering the model to skillfully discern latent snoRNA similarity patterns and comprehend the influence of higher order neighbor characteristics on the present node within the network. This pioneering approach substantially contributes to the comprehensive improvement of model performance.

**Table 2:** The results of comparative experiment

	RNADisease			independent testing		
	AUC	AUPR	accuracy	AUC	AUPR	accuracy
AMNMDA	0.6572	0.7244	0.6506	0.5010	0.4945	0.4897
NIMCGCN	0.6803	0.6655	0.6141	0.7084	0.7781	0.5205
NSAMDA	0.7023	0.7588	0.6826	0.5072	0.6726	0.6911
VGAMF	0.7304	0.7665	0.6809	0.7069	0.7049	0.6457
iPiDA_GCN	0.8101	0.8173	0.7054	0.6182	0.6185	0.6651
IGCNSDA	**0.8438**	**0.8744**	**0.7831**	**0.7142**	**0.8746**	**0.7290**

To further assess the model’s performance, we computed the average runtime for each epoch and the memory footprint during the execution of IGCNSDA, alongside five other comparative experimental methods. We run all experiments on the CPU, and the CPU used is Intel(R) Core(TM) i5-8250U CPU. The results are illustrated in Table 3. As indicated in Table 3, IGCNSDA outperforms other comparison methods in both runtime and memory utilization. Notably, its runtime is 70 times faster than the suboptimal method. Regarding memory usage, IGCNSDA consumes 0.6 times less memory than alternative methods. These findings underscore the efficacy of IGCNSDA as a valuable tool for predicting potential snoRNA–disease associations.

**Table 3:** Runtime and memory used by IGCNSDA and other methods on RNADisease dataset

	runtime(s)	memory(MB)
AMNMDA	4.7301	178.3
NIMCGCN	11.2671	198.6
NSAMDA	11.2671	180.7
VGAMF	11.6280	210.7
iPiDA_GCN	4.7300	1737.8
IGCNSDA	**0.0674**	**108.3**

#### 2.2.2 Independent test

To thoroughly assess the effectiveness of IGCNSDA, we implemented an independent testing phase. During the independent testing, all associations from the RNADisease database were utilized to train each model, and IGCNSDA’s performance was assessed using ncRPheo, as depicted in Table 2 (Independ testing). In independent testing, IGCNSDA also achieved the best predictive performance. The AUC reached 0.7142, the AUPR reached 0.8746 and the accuracy reached 0.7290, both surpassing the suboptimal method by 0.58%, 9.65% and 3.79%, respectively. This further corroborates the significant impact of IGCNSDA in improving predictive performance by partitioning the graph into different subgraphs and aggregating neighbor node information within each subgraph, thereby capturing higher order associations between snoRNA–disease nodes.

### 2.3 Noisy data sensitivity analysis

When LightGCN aggregates information from neighboring nodes, it may inadvertently involve dissimilar snoRNAs associated with common diseases, contributing to the introduction of noisy data during the embedding updating process. In contrast, IGCNSDA mitigates this issue by partitioning similar snoRNAs and their associated diseases into subgraphs, effectively isolating them from the potential influence of noise data and enhancing the robustness of experimental results. To validate this, we conducted sensitivity analysis experiments on noisy data. Initially, we introduced noise data equivalent to 1% and 5% of the total sample count into the RNADisease training and RNADisease test sets, creating four distinct datasets: RNADisease_train_1, RNADisease_train_5, RNADisease_test_1 and RNADisease_test_5. Subsequently, we utilized IGCNSDA to train and assess the model. The corresponding experimental results are presented in Table 4.

**Table 4:** The results of noisy data sensitivity analysis

	AUC	AUPR	accuracy
RNADisease	0.8438	0.8744	0.7831
RNADisease_train_1	0.8414	0.8719	0.7792
RNADisease_train_5	0.8327	0.8614	0.7725
RNADisease_test_1	0.8180	0.8476	0.7660
RNADisease_test_5	0.7827	0.8099	0.7155

The experimental results reveal that the introduction of noise data into the training set has a minimal impact on the outcomes. The AUC decreases by a maximum of 1.11%, AUPR decreases by a maximum of 1.3% and accuracy decreases by a maximum of 1.06%. This suggests that our subgraph generation algorithm has successfully achieved the anticipated effect by mitigating the influence of noisy data to a certain extent. However, when noisy data are incorporated into the test set, there is a notable decline in the model’s performance. Specifically, AUC drops by up to 6.11%, AUPR drops by 6.45% and accuracy drops by 6.76%. We hypothesize that this decline is attributed to the prevalence of noisy data in the test set, significantly affecting the model’s performance on evaluation metrics. To validate this assumption, we scrutinized the prediction results of the noisy data in the test set and observed that the prediction accuracy for noisy data in both the RNADisease_test _1 and RNADisease_test_5 datasets reached 100%. This observation underscores the processing capabilities of the IGCNSDA subgraph generation algorithm in handling noisy data.

### 2.4 Ablation experiment

**Figure 3:**
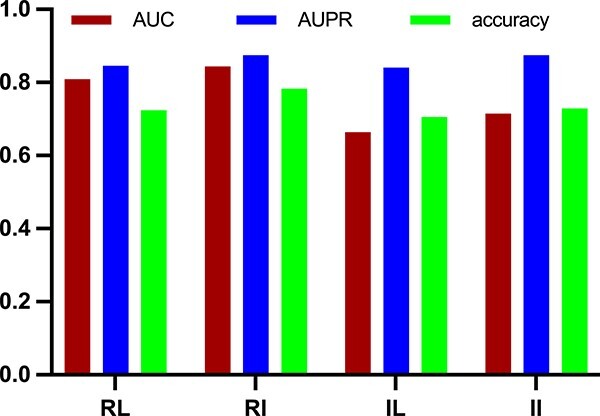
The histogram of ablation experiment. RL denotes the outcomes achieved through the application of LightGCN on the RNADisease dataset, while RI signifies the results obtained using IGCNSDA on the same dataset. IL corresponds to the results obtained using LightGCN on the indirect testing dataset, and II stands for the outcomes obtained by utilizing LightGCN on the indirect testing dataset, with results obtained by IGCNSDA.

Compared with conventional GCN algorithms, IGCNSDA employs a unique subgraph generation algorithm to aggregate similar snoRNAs, enabling IGCNSDA to learn better node embeddings. To assess the impact of the subgraph generation algorithm on model performance enhancement, we conducted ablation experiments, comparing the performance of IGCNSDA with that of LightGCN, as shown in Figure 3. Figure 3 underscores the pivotal contribution of the subgraph generation algorithm in enhancing the model’s overall performance.

### 2.5 Parameter sensitivity analysis

**Figure 4:**
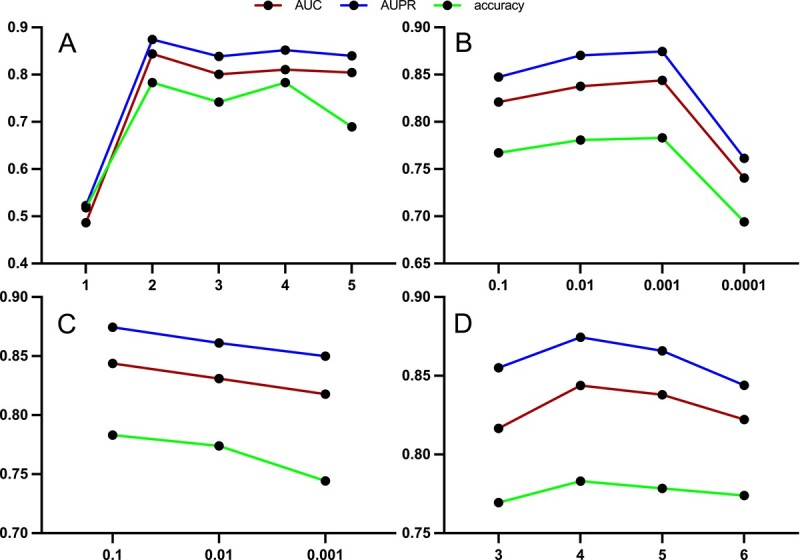
Parameter sensitivity analysis experimental results are presented as follows: (**A**) The influence of the number of LightGCN layers on experimental outcomes. (**B**) The effect of the learning rate (lr) on experimental results. (**C**) The impact of $\lambda $ on experimental results. (**D**) The influence of the number of subgraphs on experimental outcomes.

Careful parameter selection plays a crucial role in shaping the performance of IGCNSDA. Consequently, we conducted a comprehensive analysis of parameter sensitivity, focusing on four key variables: the number of LightGCN layers, learning rate (lr), $\lambda $, and the number of subgraphs. In terms of the number of layers in LightGCN, our selections ranged from [1, 2, 3, 4, 5]. For lr, we considered values of [0.1, 0.01, 0.001, 0.0001, 0.00001]. Regarding $\lambda $, we opted for [0.1, 0.01, 0.001]. For the quantity of subgraphs, we chose [3, 4, 5, 6]. The results of the parameter sensitivity analysis are illustrated in Figure 4. It is noteworthy that optimal results were achieved by configuring the number of LightGCN layers to 2 (Figure 4A), setting lr to 0.001 (Figure 4B), establishing $\lambda $ at 0.1 (Figure 4C) and specifying the number of subgraphs as 4 (Figure 4D).

### 2.6 Interpretability analysis

snoRNAs serve as integral components in various fundamental biological processes. Dysregulation in the expression of snoRNAs can lead to disruptions in both normal physiological functions and pathological processes, ultimately contributing to the initiation and progression of a diverse spectrum of diseases. It is noteworthy that snoRNAs sharing structural similarities often oversee common biochemical pathways, rendering them more likely to be associated with analogous disease manifestations. In addressing these intricate relationships, IGCNSDA integrates a subgraph generation algorithm aimed at clustering functionally similar snoRNAs and their respective disease associations into discrete subgraphs. This approach, in turn, promotes the aggregation of features among neighboring nodes within these subgraphs, thereby effectively capturing snoRNA similarity.

**Figure 5:**
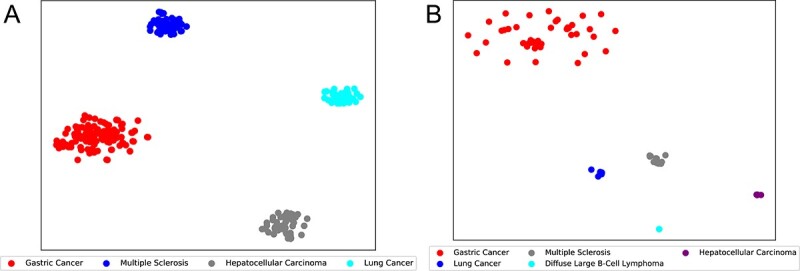
Embedding visualization results of RNADisease train set(**A**) and test set(**B**) using t-SNE.

To validate the efficacy of this methodology, we initially trained IGCNSDA to generate embeddings for all known snoRNAs and their associated diseases. We then selected four prevalent diseases, namely Gastric Cancer, Multiple Sclerosis, Hepatocellular Carcinoma and Lung Cancer, for in-depth examination. Subsequently, we concatenated the embeddings of these four diseases with the embeddings of all snoRNAs that have known associations with them in the RNADisease train dataset. We employed the t-SNE algorithm to visualize the results, as illustrated in Figure 5A. Our observations conclusively demonstrate the proficiency of IGCNSDA in effectively clustering the embeddings related to these four diseases. Furthermore, we created visual representations depicting the correlation pairs among the five diseases (Gastric Cancer, Lung Cancer, Multiple Sclerosis, Diffuse Large B-Cell Lymphoma and Hepatocellular Carcinoma) and their respective snoRNAs within the RNADisease test dataset, as depicted in Figure 5B. Our findings unequivocally highlight the efficacy of IGCNSDA in proficiently clustering the embeddings associated with these five diseases.

**Figure 6:**
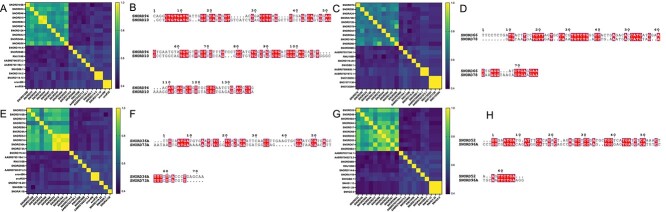
The heatmap represents the pairwise similarities of embeddings. Specifically, it focuses on comparisons between the top 10 snoRNAs, each known for well-established links to their respective diseases and the bottom 10 snoRNAs, which do not have confirmed associations with the diseases predicted by IGCNSDA. This analysis pertains to Gastric Cancer (**A**), Multiple Sclerosis (**C**), Hepatocellular Carcinoma (**E**) and Lung Cancer (**G**). The sequence alignment results for the two snoRNAs exhibiting the highest similarity in each disease group are detailed in panels **B**, **D**, **F** and **H**.

To further assess the interpretability of IGCNSDA, we identified the top 10 snoRNAs predicted by IGCNSDA with well-established links to the four diseases. Conversely, we selected the bottom 10 snoRNAs devoid of confirmed associations. Subsequently, we computed the cosine similarity of embeddings between these two sets of snoRNAs and visually represented the results in Figure 6D, F, H and J. Notably, the top 10 ranked snoRNAs exhibited significantly higher similarity scores than the last 10 snoRNAs.

For a more in-depth exploration of higher order similarities, we chose the two most closely related snoRNAs for each disease and performed an automated multiple alignment of their snoRNA sequences using the ClustalW program [41]. The outcomes were visually presented in Figure 6 with the aid of ESPript [42]. These findings furnish robust evidence in support of our perspective regarding the potential associations between snoRNA sequences and their corresponding diseases, thus reinforcing the credibility and interpretability of IGCNSDA in this context.

### 2.7 Case study

To provide robust evidence of IGCNSDA’s effectiveness, we conducted case studies focused on two prevalent human diseases: Prostate Cancer and Lung Cancer. We meticulously excluded snoRNAs specifically linked to these diseases and applied our disease-specific prediction model to the remaining snoRNAs. Subsequently, we ranked the candidate snoRNAs in descending order based on their prediction scores and selected the top 10 candidates for in-depth analysis. To evaluate their performance, we cross-referenced these top 10 candidate snoRNAs with recent literature and clinical trials retrieved from the PubMed database. The experimental results are presented in Table 5. From the experimental data, it is evident that in the case of Prostate Cancer, among the top 10 candidates, we successfully identified 9 associations that have been experimentally validated in previous studies. For example, Dong *et al.* [43] identified SnoRNA U50 (SNORD50A) as a candidate tumor suppressor gene located at 6q14.3 in their research. Mutations in this gene have been closely associated with clinically significant prostate cancer. Similarly, for Lung Cancer, among the top 10 candidates, we identified 8 associations that have undergone experimental validation in prior studies. For instance, Dong *et al.* [44] identified SNORD55 as a potential biomarker for the early diagnosis of non-small cell lung cancer in their research.

**Table 5:** Prediction results of the top 10 Prostate Cancer-associated snoRNAs (left) and top 10 Lung Cancer-associated snoRNAs (right)

**snoRNAs**	**PMID**	**snoRNAs**	**PMID**
SNORD50A	18202102	SNORD94	31338327
SNORD48	26078486	SNORD52	32802196
SNORD10	31338327	SNORD50A	26595770
SNORD105B	29554660	SNORD96A	unconfirmed
SNORD44	22788411	SNORD49A	32111002
SNORD4A	30107644	SNORD55	33474827
SNORD43	unconfirmed	SNORD43	28817650
SNORD52	32802196	SNORD68	unconfirmed
SNORD55	33474827	SNORD67	31338327
SNORD67	37492704	SNORD59A	34938414

## 3 CONCLUSIONS

Several recent studies have highlighted the crucial role of snoRNAs in the context of various diseases. Predicting the connections between snoRNAs and diseases offers valuable insights into the pathogenic mechanisms of complex human diseases, and it holds great promise for enhancing disease diagnosis and treatment. This study introduces a novel method, IGCNSDA, which utilizes an interpretable GCN for predicting snoRNA–disease associations. Our approach leverages subgraph generation algorithms to group similar snoRNAs and subsequently applies the LightGCN algorithm within each subgraph. This approach allows us to capture higher order relationships between snoRNAs and diseases while maintaining interpretability. Comprehensive experimental results unequivocally demonstrate the superior performance of our proposed method compared with recent, highly correlated approaches. Moreover, interpretability analysis provides strong evidence supporting the model’s predictions. To emphasize the practical value of IGCNSDA, we present a compelling case study that validates its effectiveness as a valuable tool for predicting potential snoRNA–disease associations in a biologically meaningful and clinically relevant manner. The conventional association prediction model functions as a black box, offering prediction outcomes without elucidating its association mechanism. In contrast, IGCNSDA stands out as an interpretable model capable of delving into the association mechanism between snoRNA–disease. It not only yields a predictable outcome but also furnishes insights that are crucial for clinical trials, mechanistic treatments and drug development, thereby enhancing controllability in predictions. Nevertheless, in the case of most models relying on GCNs, an escalation in the number of graph convolutional layers invariably leads to overfitting issues. Capitalizing on the progress in transfer learning, our forthcoming approach entails pre-training extensive models utilizing large-scale datasets. Following this, we engage in fine-tuning on downstream datasets and customize interpretable tasks according to the distinct characteristics of these datasets. This methodology seeks to bolster the model’s generalization capability, mitigate over-smoothing problems and enhance the interpretability of the model.

**Key Points**

- Accurate identification of the relationships between snoRNAs and diseases is essential for disease detection and treatment advancement. Traditional biological experimentation methods are labor-intensive, expensive and not highly scalable.
- Deep learning techniques are increasingly being used to predict snoRNA–disease associations, but they are criticized for their interpretability.
- The study introduces IGCNSDA, a GCN approach that is both innovative and interpretable for efficient snoRNA–disease association inference.
- IGCNSDA not only outperforms other methods but also provides interpretability, offering valuable insights into snoRNA–disease associations and serving as a useful tool for predicting potential associations.

## Author Biographies

**Xiaowen Hu** is a graduate student at the School of Computer, Central South University, Changsha, China. His research interests include machine learning and bioinformatics.

**Pan Zhang** is a PhD student at the Xiangya School of public health, Central South University, Changsha, China. Her research interests include viral epidemiology and bioinformatics.

**Dayun Liu** is a graduate student at the School of Computer, Central South University, Changsha, China. Her research interests include machine learning and bioinformatics.

**Jiaxuan Zhang** is a graduate student at the Department of Electrical and Computer Engineering, University of California, San Diego, United States. Her research interests include machine learning, data science and bioinformatics.

**Yuanpeng Zhang** is a graduate student at the School of Software, Xinjiang University, Urumqi, China. His research interests include machine learning and bioinformatics.

**Yanhao Fan** is a graduate student at the School of Computer, Central South University, Changsha, China. Her research interests include machine learning and bioinformatics.

**Yanhao Fan** is a graduate student at the School of Computer, Central South University, Changsha, China. Her research interests include machine learning and bioinformatics.

**Lei Deng** is a professor at the School of Computer Science and Engineering, Central South University, Changsha, China. His research interests include data mining, bioinformatics and systems biology.

## References

[1] Kiss-László Z , Henry Y, Jean-Pierre Bachellerie M, et al. Site-specific ribose methylation of preribosomal rna: a novel function for small nucleolar rnas. Cell 1996;85(7):1077–88. 8674114 10.1016/s0092-8674(00)81308-2

[2] Kiss T . Small nucleolar rna-guided post-transcriptional modification of cellular rnas. EMBO J 2001;20(14):3617–22. 11447102 10.1093/emboj/20.14.3617 PMC125535

[3] d’Orval BC , Bortolin M-L, Gaspin C, Bachellerie J-P. Box C/D RNA guides for the ribose methylation of archaeal tRNAs. The tRNATrp intron guides the formation of two ribose-methylated nucleosides in the mature tRNATrp. Nucleic Acids Res 2001;29(22):4518–29. 11713301 10.1093/nar/29.22.4518 PMC92551

[4] Zemann A , op de Bekke A, Kiefmann M, et al. Evolution of small nucleolar RNAs in nematodes. Nucleic Acids Res 2006;34(9):2676–85. 16714446 10.1093/nar/gkl359 PMC1464110

[5] Kishore S , Stamm S. The snorna hbii-52 regulates alternative splicing of the serotonin receptor 2c. Science 2006;311(5758):230–2. 16357227 10.1126/science.1118265

[6] Esteller M . Non-coding rnas in human disease. Nat Rev Genet 2011;12(12):861–74. 22094949 10.1038/nrg3074

[7] Kawalerski RR , Leach SD, Escobar-Hoyos LF. Pancreatic cancer driver mutations are targetable through distant alternative rna splicing dependencies. Oncotarget 2021;12:525–33. 33796221 10.18632/oncotarget.27901 PMC7984828

[8] Zheng D , Zhang J, Ni J, et al. Small nucleolar rna 78 promotes the tumorigenesis in non-small cell lung cancer. J Exp Clin Cancer Res 2015;34(1):1–15. 25975345 10.1186/s13046-015-0170-5 PMC4472183

[9] Krishnan P , Ghosh S, Wang B, et al. Profiling of small nucleolar rnas by next generation sequencing: potential new players for breast cancer prognosis. PLoS One 2016;11(9):e0162622. 10.1371/journal.pone.0162622 PMC5025248 27631501

[10] Okugawa Y , Toiyama Y, Toden S, et al. Clinical significance of snora42 as an oncogene and a prognostic biomarker in colorectal cancer. Gut 2017;66(1):107–17. 26475630 10.1136/gutjnl-2015-309359 PMC4860159

[11] Liao J , Lei Y, Mei Y, et al. Small nucleolar rna signatures as biomarkers for non-small-cell lung cancer. Mol Cancer 2010;9(1):1–10. 20663213 10.1186/1476-4598-9-198 PMC2919450

[12] Chen J , Lin J, Hu Y, et al. RNADisease v4.0: an updated resource of RNA-associated diseases, providing RNA-disease analysis, enrichment and prediction. Nucleic Acids Res 2023;51(D1):D1397–404. 36134718 10.1093/nar/gkac814 PMC9825423

[13] Zhang W , Yao G, Wang J, et al. Ncrpheno: a comprehensive database platform for identification and validation of disease related noncoding rnas. RNA Biol 2020;17(7):943–55. 32122231 10.1080/15476286.2020.1737441 PMC7549653

[14] Deng L , Chen Z. An integrated framework for functional annotation of protein structural domains. IEEE/ACM Trans Comput Biol Bioinform 2015;12(4):902–13. 26357331 10.1109/TCBB.2015.2389213

[15] Shi Z , Zhang H, Jin C, et al. A representation learning model based on variational inference and graph autoencoder for predicting lncrna-disease associations. BMC Bioinform 2021;22(1):1–20. 10.1186/s12859-021-04073-z PMC7983260 33745450

[16] Zhu R , Wang Y, Liu J-X, Dai L-Y. Ipcarf: improving lncrna-disease association prediction using incremental principal component analysis feature selection and a random forest classifier. BMC Bioinform 2021;22(1):1–17. 10.1186/s12859-021-04104-9 PMC8017839 33794766

[17] Wang M-N , You Z-H, Wang L, et al. Ldgrnmf: Lncrna-disease associations prediction based on graph regularized non-negative matrix factorization. Neurocomputing 2021;424:236–45.

[18] Fan Y , Chen M, Pan X. Gcrflda: scoring lncrna-disease associations using graph convolution matrix completion with conditional random field. Brief Bioinform 2022;23(1):bbab361. 34486019 10.1093/bib/bbab361

[19] Deng L , Li W, Zhang J. Ldah2v: exploring meta-paths across multiple networks for lncrna-disease association prediction. IEEE/ACM Trans Comput Biol Bioinform 2019;18(4):1572–81. 10.1109/TCBB.2019.2946257 31725386

[20] Chen X , Li T-H, Zhao Y, et al. Deep-belief network for predicting potential mirna-disease associations. Brief Bioinform 2021;22(3):bbaa186. 34020550 10.1093/bib/bbaa186

[21] Jin C , Shi Z, Lin K, Zhang H. Predicting mirna-disease association based on neural inductive matrix completion with graph autoencoders and self-attention mechanism. Biomolecules 2022;12(1):64. 35053212 10.3390/biom12010064 PMC8774034

[22] Zhang Y , Lei X, Pan Y, Pedrycz W. Prediction of disease-associated circrnas via circrna–disease pair graph and weighted nuclear norm minimization. Knowl-Based Syst 2021;214:106694.

[23] Lei X-J , Bian C, Pan Y. Predicting circrna-disease associations based on improved weighted biased meta-structure. J Comput Sci Technol 2021;36(2):288–98.

[24] Wang L , You Z-H, Li Y-M, et al. Gcncda: a new method for predicting circrna-disease associations based on graph convolutional network algorithm. PLoS Comput Biol 2020;16(5):e1007568. 10.1371/journal.pcbi.1007568 PMC7266350 32433655

[25] Ji B , Luo J, Pan L, et al. Dfl-pida: prediction of piwi-interacting rna-disease associations based on deep feature learning. In: 2021 IEEE International Conference on Bioinformatics and Biomedicine (BIBM), pp. 406–11. Houston, TX, USA: IEEE, 2021.

[26] Zheng K , Zhang XL, Wang L, et al. SPRDA: a link prediction approach based on the structural perturbation to infer disease-associated piwi-interacting RNAs. Brief Bioinform 2023;24(1):bbac498. 10.1093/bib/bbac498 36445194

[27] Sun Z , Huang Q, Yang Y, et al. PSnoD: identifying potential snoRNA-disease associations based on bounded nuclear norm regularization. Brief Bioinform 2022;23(4):bbac240. 10.1093/bib/bbac240 35817303

[28] Wang X , He X, Wang M, et al. Neural graph collaborative filtering. In: Proceedings of the 42nd International ACM SIGIR Conference on Research and Development in Information Retrieval (SIGIR'19). Association for Computing Machinery, New York, NY, USA, 2019; 165–174. 10.1145/3331184.3331267.

[29] He X , Deng K, Wang X, et al. Lightgcn: simplifying and powering graph convolution network for recommendation. In: Proceedings of the 43rd International ACM SIGIR Conference on Research and Development in Information Retrieval (SIGIR'20). Association for Computing Machinery, New York, NY, USA, 2020; 639–648. 10.1145/3397271.3401063.

[30] Yupeng H , Zhan P, Yang X, et al. Temporal representation learning for time series classification. Neural Comput Appl 2021;33:3169–82.

[31] Rendle S . Factorization machines. In: ICDM 2010, The 10th IEEE International Conference on Data Mining, Sydney, Australia, 14–17 December 2010, 2010.

[32] He X , Zhang H, Kan MY, Chua TS. Fast matrix factorization for online recommendation with implicit feedback. In: Proceedings of the 39th International ACM SIGIR conference on Research and Development in Information Retrieval (SIGIR'16). Association for Computing Machinery, New York, NY, USA, 2016, 549–558. 10.1145/2911451.2911489.

[33] Dao F-Y , Lv H, Fullwood MJ, Lin H. Accurate identification of dna replication origin by fusing epigenomics and chromatin interaction information. Research 2022;2022; 9780293. 10.34133/2022/9780293 PMC9667886 36405252

[34] Zou X , Ren L, Cai P, et al. Accurately identifying hemagglutinin using sequence information and machine learning methods. Front Med (Lausanne) 2023;10:1281880. 38020152 10.3389/fmed.2023.1281880 PMC10644030

[35] Li J , Zhang S, Liu T, et al. Neural inductive matrix completion with graph convolutional networks for miRNA-disease association prediction. Bioinformatics 2020;36(8):2538–46. 31904845 10.1093/bioinformatics/btz965

[36] Ning Q , Zhao Y, Gao J, et al. AMHMDA: attention aware multi-view similarity networks and hypergraph learning for miRNA-disease associations identification. Brief Bioinform 2023;24(2):bbad094. 10.1093/bib/bbad094 36907654

[37] Zhao H , Li Z, You ZH, et al. Predicting Mirna-disease associations based on neighbor selection graph attention networks. IEEE/ACM Trans Comput Biol Bioinform 2023;20(2):1298–307. 36067101 10.1109/TCBB.2022.3204726

[38] Hou J , Wei H, Liu B. iPiDA-GCN: identification of piRNA-disease associations based on graph convolutional network. PLoS Comput Biol 2022;18(10):e1010671. 36301998 10.1371/journal.pcbi.1010671 PMC9662734

[39] Ding Y , Lei X, Liao B, Wu FX. Predicting miRNA-disease associations based on multi-view variational graph auto-encoder with matrix factorization. IEEE J Biomed Health Inform 2022;26(1):446–57. 34111017 10.1109/JBHI.2021.3088342

[40] Yang H , Luo YM, Ma CY, et al. A gender specific risk assessment of coronary heart disease based on physical examination data. NPJ Digit Med 2023;6(1):136. 37524859 10.1038/s41746-023-00887-8 PMC10390496

[41] Thompson JD , Gibson TJ, Higgins DG. Multiple sequence alignment using clustalw and clustalx. Curr Protoc Bioinform 2003;00; Chapter 2:Unit 2.3. 10.1002/0471250953.bi0203s00 18792934

[42] Robert X , Gouet P. Deciphering key features in protein structures with the new endscript server. Nucleic Acids Res 2014;42(W1):W320–4. 24753421 10.1093/nar/gku316 PMC4086106

[43] Dong XY , Rodriguez C, Guo P, et al. SnoRNA U50 is a candidate tumor-suppressor gene at 6q14.3 with a mutation associated with clinically significant prostate cancer. Hum Mol Genet 2008;17(7):1031–42. 18202102 10.1093/hmg/ddm375 PMC2923223

[44] Dong X , Song X, Ding S, et al. Tumor-educated platelet SNORD55 as a potential biomarker for the early diagnosis of non-small cell lung cancer. Thorac Cancer 2021;12(5):659–66. 33474827 10.1111/1759-7714.13823 PMC7919130

